# Cardiological Aspects of Feeding and Eating Disorders in Children and Adolescents and Associations with Refeeding Syndrome, Purging Behaviors, and Psychoactive Drugs

**DOI:** 10.3390/jcdd12020066

**Published:** 2025-02-10

**Authors:** Chiara Letizia, Jacopo Pruccoli, Umberto Pannacci, Tania Napolitano, Marianna Fabi, Antonia Parmeggiani

**Affiliations:** 1 IRCCS Istituto delle Scienze Neurologiche di Bologna, UOC Neuropsichiatria dell’Età Pediatrica, Centro Regionale per i Disturbi della Nutrizione e dell’Alimentazione in Età Evolutiva, 40139 Bologna, Italy; 2 Dipartimento di Scienze Mediche e Chirurgiche (DIMEC), Università di Bologna, 40126 Bologna, Italy; 3 Pediatric Emergency Unit, IRCCS Azienda Ospedaliero-Universitaria di Bologna, 40139 Bologna, Italy

**Keywords:** Feeding and Eating Disorders, developmental age, electrocardiographic alterations, Anorexia Nervosa, Bulimia Nervosa, Refeeding Syndrome, cardiac complications

## Abstract

Feeding and Eating Disorders (FEDs) constitute a complex spectrum of psychiatric conditions, impacting physical and psychosocial well-being. This retrospective observational study aimed to dissect the electrocardiographic (ECG) alterations in pediatric patients with FEDs, correlating them with clinical factors, treatment modalities, Refeeding Syndrome (RS) and the reversibility of ECG abnormalities post-treatment. Analyzing records from a third level Italian Regional Center for FEDs in children and adolescents, the study encompassed 150 patients meeting the inclusion criteria. Sinus bradycardia was the prevalent ECG alteration, notably in Anorexia Nervosa (AN) restrictive type. Association analyses revealed links between the severity of AN, hormonal imbalances, and amenorrhea and ECG abnormalities. Pharmacological interventions, particularly antipsychotics, exhibited associations with a QT interval prolongation. RS demonstrated significant correlations with potassium and magnesium imbalances, which were linked to specific ECG changes. This study highlighted the reversibility of ECG abnormalities, concomitant with Body Mass Index improvement. This analysis underscores the critical cardiac implications of FEDs, advocating for multidisciplinary interventions and close cardiac monitoring. Early detection and holistic care are imperative in managing patients with FEDs in the developmental age, offering potential reversibility of cardiac alterations post-treatment. These findings underscore the need for prospective studies to validate these observations and delve deeper into cardiac involvement in FEDs.

## 1. Introduction

Feeding and Eating Disorders (FEDs) constitute psychiatric conditions characterized by disrupted eating behaviors, adversely affecting physical health and psychosocial well-being [1]. The Diagnostic and Statistical Manual of Mental Disorders (DSM-5) outlines six primary diagnostic categories, with Anorexia Nervosa (AN), Bulimia Nervosa (BN), and Binge-Eating Disorder (BED) being the most prevalent. Conditions such as Avoidant-Restrictive Food Intake Disorder (ARFID), Pica, and Rumination Disorder, once predominantly observed in childhood, are now considered part of the same spectrum [2]. There are also two residual categories: Other Specified Feeding and Eating Disorders (OSFEDs) and Unspecified Feeding or Eating Disorders (UFEDs).

The impact of eating disorders on the youth population is significant, with a lifetime prevalence among women under 30 ranging from 5.5% to 17.9% and from 0.6% to 2.4% in men, with a minimum age of 9 years [3].

FEDs exhibit some of the highest fatality rates among mental health disorders, particularly AN, which has a standardized mortality ratio of 5.86 [4]. The causes of death are due to health issues caused by the disorder, and one in five deaths are caused by suicide [5].

The medical complications arising from FEDs stem from malnutrition, weight loss, and purging behaviors, affecting various organs and bodily systems [6]. While most complications are reversible with timely and effective treatment, some can lead to permanent damage or potential life-threatening situations, such as cardiac complications that can lead to sudden death [7,8].

Cardiac structural and functional changes result from severe weight loss due to malnutrition, impacting hemodynamics and electrical activity [9]. Reduced left ventricular mass, dimensions, stroke volume, cardiac output, and cardiac index have been observed in cases of myocardial atrophy due to hyponutrition [9]. AN patients frequently exhibit mitral valve prolapse and pericardial effusion, which are typically reversible with weight restoration [10].

The literature highlights electrocardiographic alterations and associated arrhythmic risks in FEDs. Sinus bradycardia, prevalent in AN patients, is attributed to increased vagal tone, reduced energy metabolism, and compensatory mechanisms against cardiac failure [11]. A prolonged QT interval, associated with torsade de pointes and sudden cardiac death, is often linked with factors such as hypokalemia, hypomagnesemia, and certain medications, rather than being intrinsic to Anorexia Nervosa [10].

Electrocardiographic changes encompass alterations in the QRS complex, such as a reduction in its amplitude and right-axis deviation, notably in AN, which suggests a reduced left ventricular mass [8,12]. A decreased mean R wave amplitude was also found in both AN and BN patients [13,14]. Atrial conduction abnormalities and atrioventricular defects are described less commonly [15,16].

Most electrocardiographic changes tend to resolve with body weight restoration, though close monitoring is essential due to the risk of Refeeding Syndrome-induced electrolyte imbalances, leading to cardiac dysfunction [17,18].

Despite the significant impact of these alterations, there has been limited focus on patients in the developmental age. Thus, this retrospective study endeavors to identify electrocardiographic changes in children and adolescents with FEDs, correlate these alterations with associated factors, and assess their reversibility post-treatment.

## 2. Materials and Methods

### 2.1. Study Design and Participants

This retrospective observational study was conducted using data gathered from a third level Italian Regional Center for FEDs in children and adolescents. Ethical approval for this study (code ECG-DCA-2020) was obtained from the local ethics committee.

This study was carried out in March 2023, retrospectively analyzing the records of patients admitted to the study center between 1 October 2014, and 31 March 2020. Hospitalization encompassed both inpatient and day hospital (DH) treatments. The DH treatment program provided to FED patients was structured similarly to inpatient care and was equally intensive. The multidisciplinary intervention offered at our Center consisted of psychological, psychopharmacological, and nutritional components, following international clinical guidelines. All treatment groups adhered to the same comprehensive program delivered by a unified team within the same Center.

Inclusion criteria comprised patients aged 10 to 18 years with either a new or previously diagnosed FED based on specific DSM-5 criteria [1]. Patients with known pre-existing cardiovascular conditions were excluded.

### 2.2. Evaluation Procedures

During hospitalization, patients underwent diagnostic and instrumental procedures in line with clinical FED guidelines. This comprehensive program encompassed psychopathological, nutritional, biochemical, and, if needed, instrumental screenings.

In addition to electrocardiographic data, the variables considered included demographics (such as gender and age), type of FED diagnosis, associated clinical characteristics (such as BMI, subtype, type and frequency of purging behaviors and binge-eating behaviors, presence of excessive exercise, amenorrhea, and comorbidities), duration of untreated illness, and duration of hospitalization. Diagnosis of FED and comorbidities was conducted by neuropsychiatrists and clinical psychologists specialized in FED, adhering to DSM-5 diagnostic criteria [1]. Mood deviation was assessed through the SAFA (Self-Administered Psychiatric Scales for Children and Adolescents), an Italian psychometric test.

Electrocardiographic data were obtained via standard 12-lead electrocardiograms (ECGs) performed while the patient was at rest, following standard parameters. A pediatric cardiology specialist (M.F.) reviewed the collected ECG tracings, calculated the presence of sinus bradycardia (HR < 60 bpm), and marked sinus bradycardia (<50 bpm), tachycardia (HR > 100 bpm), and PR interval prolongation (≥200 ms), and identified first degree atrioventricular block and QRS complex prolongation (>120 ms). The corrected QT interval (QTc) was calculated using appropriate formulas and considered pathological if exceeding 440 ms (borderline between 440 ms and 460 ms, extended >460 ms).

When available, echocardiogram reports were also compiled. Laboratory values included electrolyte levels and thyroid hormone levels (TSH, fT3, and fT4). Psychopharmacological therapy details were also recorded.

Three separated time frames were considered across the duration of the hospitalization: admission (T0), a middle phase halfway between T0 and the last visit at discharge (T1), and discharge (T2), according to available data.

All the variables described above were collected at T0 and at T2. Some of the variables, such as the ECGs, the echocardiogram, laboratory values, and psychopharmacological therapy, were collected also in the middle phase of hospitalization (T1).

Refeeding Syndrome (RS) presence was assessed using ASPEN criteria [19], considering electrolyte changes during refeeding. Specifically, the blood samples considered assessed potassium (K), magnesium (Mg), and phosphorus (P) concentrations at hospitalization (T0) and at the second check-up during refeeding (T1). This extensive assessment aimed to identify and correlate electrocardiographic changes in pediatric FED patients while examining associated factors and assessing reversibility post-treatment.

In particular, to understand whether clinical, biological, and pharmacological features related to FEDs were associated with a higher risk of ECG alterations, a comparison was made between the group of patients who had at least one ECG alteration at admission (T0) and those who had no alterations. In addition, attention was focused on antipsychotic and antidepressant therapies and if the insertion of new therapies may be correlated with ECG changes. Finally, we investigated how purging behaviors and the occurrence of RS could impact the electrocardiogram.

### 2.3. Statistical Analysis

The participant data collected in the Data Collection Form were transcribed into Excel files and used anonymously. All analyses conducted were exploratory, and no hypotheses were formulated for testing. Means and standard deviations were provided for continuous variables, and absolute values and percentages were provided for nominal variables. Descriptive investigations were carried out on the sample as a whole. Student’s *t*-test was employed to compare continuous variables (Mann–Whitney test for non-parametric variables). The chi-square test was used to compare nominal variables (Fisher’s exact test when necessary). Shapiro–Wilk and Levene tests were used to assess data normality and variance homogeneity.

The relationship between the reversibility of waveform changes and the variation in BMI between admission and discharge was analyzed using a logistic regression model.

To assess the impact of potential factors (RS, psychopharmacological interventions, purging behaviors) on ECG changes during hospitalization, analyses of covariance (ANCOVA) were conducted. Specifically, ECG parameters such as HR, PR, QRS, QT, and QTc were considered as dependent variables. The following were considered as covariates:

- Incidence of RS (overall and for the individual electrolytes potassium, magnesium, and phosphate);
- Administration of Second-Generation Antipsychotic (SGA) (overall and for the individual SGAs olanzapine, aripiprazole, risperidone, and quetiapine) or antidepressants (overall and for the individual antidepressants Sertraline, Fluoxetine, and Fluvoxamine);
- Frequency of purging behaviors.

These ANCOVAs were ultimately adjusted using the respective baseline ECG variables (HR, PR, QRS, QT, and QTc) as covariates. The significance level was set to 0.05 for all analyses. Jeffrey’s Statistical Program (JASP), version 17.0 for Windows, was used for all investigations.

## 3. Results

### 3.1. Enrollment and Sample’s Characteristics

This study cohort comprised 160 patients with FEDs who sought treatment at our Regional Center within the designated time frame and met the inclusion criteria. However, 10 patients were excluded from the statistical analysis due to missing EEG data upon admission. Therefore, a total of 150 patients were included in the final analysis, constituting 144 females (96%) and 6 males (4%) and yielding a median age of 15.6 (IQR = 3.0; min = 10; max = 18) years.

Among the included patients, 123 (82%) were admitted as inpatients, while the remaining received DH treatment. The predominant FED type observed in the sample was AN restrictive type (N = 117, 78%), followed by AN binge-eating/purging type (N = 16, 10.7%), BN (N = 7, 4.7%), ARFID (N = 5, 3.4%), BED (N = 2, 1.3%), and 3 cases of Unspecified Feeding or Eating Disorder (UFED).

The median duration of illness was 12 (IQR = 11.0; min = 1; max = 60) months at admission. The median duration of hospitalization (T0–T2 interval) was 100.5 (IQR = 77.3; min = 9; max = 472) days. The median time between the first and the central ECG (T0–T1 interval) was 50 (IQR = 48.0; min = 6; max = 394) days. The median time between the central and the last ECG (T1–T2 interval) was 66 (IQR = 56.8; min = 7; max = 407) days.

Table 1 provides a summary of the clinical characteristics associated with FEDs and outlines the psychopharmacological treatment administered upon admission.

Table 2 shows BMI at admission for each FED type.

### 3.2. Electrocardiographic Findings in the Three Phases of Recovery

**Figure 1:**
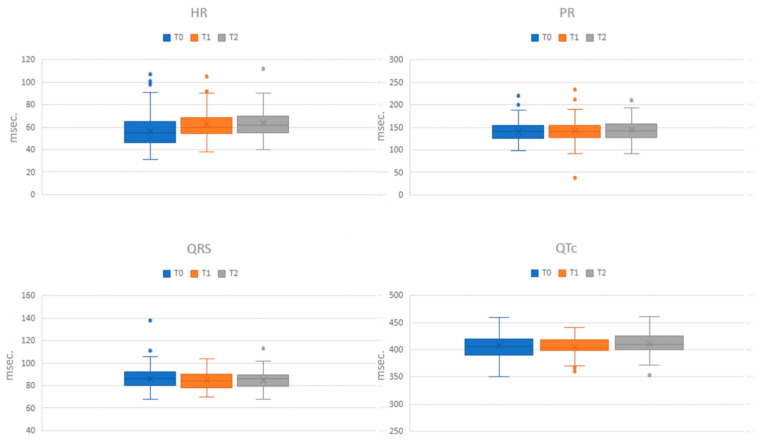
Heart rate (HR) and electrocardiographic intervals in the three phases of recovery (T0, T1, T2).

Table 3 and Figure 1 display the mean values of the electrocardiographic data across the different phases of assessment (T0, T1, and T2). Upon admission (T0), ECG alterations were observed in 114 out of 150 patients (76%). The most prevalent alteration was sinus bradycardia, identified in 98 patients (65.3%), with 51 (34%) exhibiting a heart rate (HR) below 50 bpm. Sinus tachycardia was present in three patients (2%). Additionally, two patients displayed a junctional rhythm, and one patient exhibited a junctional escape beat. Two cases of first-degree atrioventricular (AV) block and one instance of prolonged QRS tract (138 ms), indicating an interventricular conduction disorder, were recorded. Eight patients (5.3%) had QTc values ranging between 440 ms and 460 ms. A comparative analysis of the ECG alterations among the principal disorders revealed rhythm frequency alterations across all disorders, with marked sinus bradycardia notably more prevalent in AN restrictive type (*p* = 0.028). Other ECG alterations were exclusively found in AN.

In the middle phase (T1), 95 out of 150 patients underwent an ECG assessment. Sinus bradycardia was the most common alteration, identified in 41 patients (43.2%), with 12 (12.6%) having an HR below 50 bpm. Sinus tachycardia was found in one patient. Supraventricular ectopic beats were observed in one patient, while another patient displayed an ectopic atrial rhythm. Three patients exhibited first-degree AV block, and only one patient had a QTc above 440 ms (441 ms).

Upon discharge (T2), 84 out of 150 patients underwent an ECG evaluation. Sinus bradycardia remained the predominant alteration, present in 30 patients (35.7%), with 8 exhibiting severe bradycardia. Sinus tachycardia was observed in one patient and an accelerated junctional rhythm in another. One patient showed first-degree AV block. Notably, four patients had QTc values higher than 440 ms, with one extending beyond (461 ms).

### 3.3. Other Medical Variables Collected During Hospitalization

**Figure 2:**
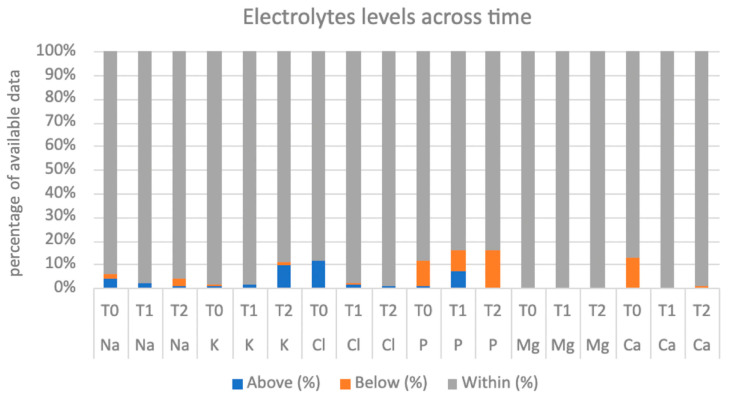
Electrolyte levels in the three phases of recovery (T0, T1, T2).

Table 4 and Figure 2 show the values of electrolytes and thyroid hormones available at the three phases of hospitalization.

Echocardiographic data performed in the three phases were also collected. At T0, 18 patients (12%) had an echocardiography. Pericardial effusion was found in five patients (four mild, one moderate) and mitral valve prolapse in three patients (two mild and one moderate). At T1, 17 patients had their first echocardiography, and 5 showed mild pericardial effusion (29.4%). At T2, five patients underwent an echocardiogram, and mild pericardial effusion was found to be already present in four cases (three at T0 and one at T1).

Regarding psychopharmacological therapy, at T0, 120 patients (80%) were taking at least one drug (drug classes at T0 are shown in Table 1). At T1, data were available for 101 patients: 96 (87.2%) were receiving psychopharmacological therapy (90% antidepressants, 64.4% antipsychotics, 5.9% mood stabilizers, and 3% benzodiazepines). At T2, 144/150 (96%) were taking pharmacological therapy: 88% antidepressants, 60% antipsychotics, 10% mood stabilizers, and 3.3% benzodiazepines.

### 3.4. Electrocardiographic Abnormalities and Associated Variables

Patients who had at least one ECG alteration at T0 were compared with the group of patients without. The severity of AN, i.e., BMI values (*p* = 0.038), reduced fT3 values (*p* = 0.003), and the presence of secondary amenorrhea (*p* = 0.003), was significantly associated with ECG alterations. The same variables were significantly different (all exhibit *p* < 0.001) when comparing patients with and without sinus bradycardia at T0.

At T2, ECG alterations were significantly associated with the presence of secondary amenorrhea (*p* = 0.002).

Looking deeper into psychopharmacological treatments—specifically second-generation antipsychotics (SGAs) and SSRIs—we analyzed the therapeutic changes during the three phases of recovery and the ECG abnormalities. The addition of new SGAs (Olanzapine, Aripiprazole, and Risperidone) in the early phase of recovery was not associated with ECG alterations. In the final phase of hospitalization, the addition of new SGAs (Olanzapine, Aripiprazole, Risperidone, and Quetiapine) was associated with a significant increase in QRS duration (151.4 ± 156.4 ms versus 85.0 ± 8.2 ms, *p* = 0.003). We did not find any association with a specific antipsychotic. The addition of new SSRIs (Sertraline, Fluoxetine, and Fluvoxamine) was not associated with ECG alterations in any phase of the hospitalization.

Finally, purging behaviors and recurrent vomiting episodes, i.e., at least one episode a week, were not significantly associated with HR (*p* = 0.603), PR (*p* = 0.119), QRS (*p* = 0.403), QT (*p* = 0.853), or QTc (*p* = 0.165) values.

### 3.5. Electrocardiographic Abnormalities and Refeeding Syndrome

In our cohort, 35/124 patients (28.2%) developed RS, following the ASPEN criteria. Considering K values, 20/124 patients (16.1%) showed RS related to potassium imbalance (17 mild RS, 2 moderate RS, and 1 case of severe RS). When considering Mg values, 5 patients out of 121 available samples (4.1%) showed RS related to magnesium imbalance (4 mild RS and 1 moderate RS). Considering P values, 14 patients out of 121 available samples (11.6%) showed RS related to phosphorus imbalance (12 mild RS and 2 moderate RS). The patients who experienced RS related to a magnesium imbalance showed a significantly prolonged PR interval (*p* = 0.009) between the ECGs at T0 and at T1 (PR = 193.3 ± 35.2 ms) compared to those who did not (PR = 142.7 ± 28.3 ms). The patients with RS related to phosphorus disequilibrium showed a significantly higher HR (*p* = 0.027) between the ECGs at T0 and at T1 (HR = 78.0 ± 28.1 bpm) than those who did not exhibit this condition (HR = 62.6 ± 11.2 bpm).

### 3.6. Reversibility of Electrocardiographic Abnormalities After Refeeding

**Figure 3:**
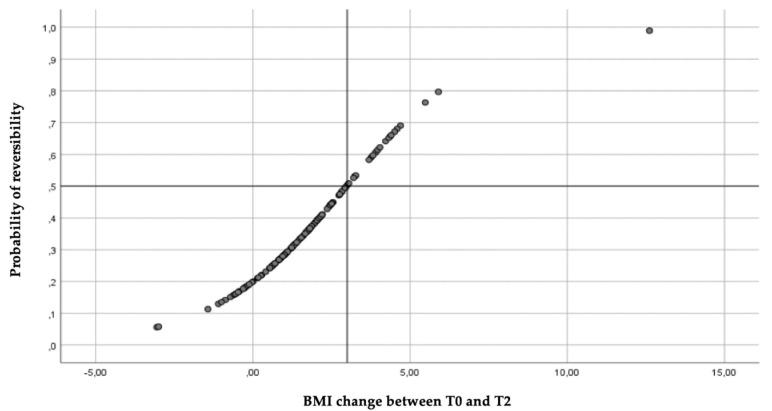
Probability of reversibility of ECG alterations as a function of BMI change between admission (T0) and discharge (T2).

A logistic regression model was built to study the relationship between the variation of BMI at T0–T2 and ECG abnormalities. The increase in BMI was significantly associated with the reversibility of ECG alterations (*p* = 0.009). For each point of BMI increase, the odds ratio of reversibility increased by 59.5% [OR: 1.595 (1.126–2.261)]. The probability that ECG alterations were reversible after RS was 50% higher if the BMI increased by at least three points (Figure 3).

## 4. Discussion

The present retrospective study underscores the cardiac implications associated with FEDs, emphasizing their substantial impact not only on psychological but also on physical health. This comprehensive retrospective analysis aimed to elucidate the ECG alterations in a sample of pediatric and adolescent patients with FEDs, correlating these changes with clinical factors, pharmacological interventions, and RS and studying their evolution after treatment. Through the study of these associations and the evolution of ECG parameters, we intended to underscore several implications of this exam in FEDs as a possible tool to monitor the severity and evolution of the condition, evaluate the treatment efficacy, and ensure the safety of psychopharmacological therapy.

Given the prevalence of FEDs, particularly among adolescents, which has been increasing in recent years, and their potential severe complications [4,5], it is urgent to identify easy tools to follow the diseases. The medical complications arising from FEDs, notably cardiac complications such as arrhythmias leading to sudden death, underscore the need for early detection and intervention in managing these conditions. An ECG is non-invasive, non-expansive, easy to administer and interpret, and feasible at the patient’s bed; thus, it could represent a valid tool for understanding the associated risks and complications.

As expected, ECGs across the different phases of recovery found a higher prevalence of alterations in over three-quarters of the patients at admission, confirming the impact of FEDs on cardiac electrical activity, especially when the patients are hospitalized (which is supposed to align with the worst status), which is in line with the literature [20]. Our findings confirm previous data showing sinus bradycardia as the most common alteration. Importantly, sinus bradycardia was more prevalent in AN restrictive type, with a significant difference from BN and BED due to the presence of marked sinus bradycardia, suggesting a potential correlation between specific FED types and ECG alterations. These findings resonate with the existing literature, emphasizing the significance of ECG abnormalities in FEDs and attributing them to various physiological and metabolic changes secondary to malnutrition and compensatory mechanisms [10]. In addition, the high frequency of sinus bradycardia in our sample, compared with a relatively short duration from the onset of symptoms to the time of admission (13.5 ± 9.5 months), confirms how this alteration represented an early sign of the disorder.

Other ECG alterations, such as sinus arrhythmias and conduction disturbances, showed low relevance in our sample, expanding the previous literature [12]. Notably, the QTc prolongation, commonly associated with eating disorders as a possible cause of increased risk of cardiovascular death [9,10], was present in our cohort at admission in 5.3% of cases. This trend of QTc values appeared to be variable during hospitalization. At discharge, 4.8% of the sample had an elongated QTc. In half of the cases, it was already present at T0 but not at T1, and only one case presented a very pathological value (461 ms), although in the absence of a clinical correlate. This finding seems to be in line with the hypothesis that QTc prolongation is not an inherent characteristic of the disorder but is probably associated with extrinsic factors, such as Ikr-inhibiting medications and electrolyte imbalances, as evidenced by Krantz and colleagues in their study involving a large cohort of adult patients [21].

This study delves into the associations between ECG alterations and clinical variables, highlighting the severity of AN, lower fT3 levels, and secondary amenorrhea as significant factors linked with ECG abnormalities, particularly sinus bradycardia (the most common), on admission. In addition, ECG anomalies persisted over time in those patients with lasting amenorrhea, suggesting that this could be a marker of severe feeding disorders impacting cardiac status.

Regarding drug intervention, our data show that SSRIs administered during hospitalization (i.e., Sertraline, Fluoxetine, and Fluvoxamine) never impacted the ECG, confirming the low cardiovascular risk profile of these specific drugs within the SSRI class. On the other hand, SGAs did not alter the ECG in the most severe stage of the disease, i.e., at admission, but they seemed to have an effect in later stages, warranting cautious and strict monitoring.

RS emerged as a critical factor, with phosphorus and magnesium imbalances significantly associated with ECG changes. In particular, RS related to phosphorus imbalances caused higher heart rates, whereas RS associated with magnesium imbalances led to PR interval prolongation. The analyzed time frame was from T0 to T1, confirming the first weeks of refeeding as the most vulnerable, necessitating meticulous surveillance of electrolyte values and electrocardiograms to promptly recognize the emergence of RS and avoid potentially dangerous outcomes.

Moreover, our findings show the reversibility of ECG alterations along with BMI improvement, highlighting the central role of nutritional rehabilitation in mitigating cardiac complications and thus morbidity and mortality.

A strength of our study is the large cohort of pediatric patients and the reliability of a large amount of data. However, the limitations are represented by its retrospective nature, resulting in incomplete data, variability in the timelines examined, a sample of FED categories that are not homogenously represented, and the absence of a control group.

## 5. Conclusions

In conclusion, this study elucidated the intricate relationship between FEDs and cardiac complications, emphasizing the need for multifaceted interventions targeting both nutritional rehabilitation and cardiac monitoring. The ECG was highlighted as an important marker for many pathological aspects due to its simplicity, minimal invasiveness, cost-effectiveness, and ease of bedside administration. Specifically, the ECG proved to be a valuable tool in assessing the severity of the disorder, evaluating the effectiveness of treatment—particularly in relation to a BMI increase and its association with secondary amenorrhea—and monitoring the safety profile of the medications administered, which requires meticulous oversight. Further prospective studies are warranted to validate these findings and explore additional nuanced aspects of cardiac implications in FEDs.

## Figures and Tables

**Table 1:** Clinical futures of the sample at admission (T0). Values are presented as mean ± standard deviation or number (%).

Variable (T0)	Total (n = 150)
BMI (kg/m^2^)	15.0 ± 3.0
Purging behaviors Binge-eating behaviors Excessive exercise Amenorrhea: • Primary • Secondary Depressed mood Antidepressants: • Selective serotonin reuptake inhibitors (SSRIs) - Sertraline - Fluoxetine - Fluvoxamine - Citalopram • Serotonin-norepinephrine reuptake inhibitors (SNRIs) Antipsychotics: • Olanzapine • Risperidone • Aripiprazole • Quetiapine Benzodiazepines Mood stabilizers	27 (18%) 22 (14.7%) 34 (51%) 23 (16%) 78 (54,1%) 116 (77.3%) 85 (56.7%) 66 (44%) 16 (10.7%) 2 (1.3%) 1 (0.7%) 2 (1.3%) 16 (10.7%) 7 (4.7%) 6 (4,0%) 1 (0.7%) 3 (2.0%) 1 (0.7%)

**Table 2:** BMI at admission for each FED type. Values are presented as mean ± standard deviation.

FED type	BMI
AN restrictive type	14.2 ± 1.8
AN binge-eating/purging type	16.7 ± 3.1
BN	21.4 ± 2.9
ARFID	13.1 ± 1.2
BED	23.9 ± 3.9
UFED	23.5 ± 10.9

**Table 3:** Electrocardiographic data in the three phases of recovery. Values are presented as mean ± standard deviation.

Electrocardiographic Data (ms)	T0 (n = 150)	T1 (n = 95)	T2 (n = 84)
HR	56.4 ± 13.7	62.6 ± 12.4	63.6 ± 11.8
PR	142.2 ± 21.0	143.7 ± 25.8	145.2 ± 21.2
QRS	85.9 ± 9.7	84.4 ± 8.5	84.5 ± 7.8
QTc	406.7 ± 22.9	404.8 ± 17.8	411.5 ± 19.0

**Table 4:** Values of electrolytes and thyroid hormones in the three phases of recovery. Continuous variables are reported as means and standard deviations; percentages of values below or over the reference value (r.v.) are reported as well.

Medical Variables	T0			T1			T2		
	Mean (SD)	Above r.v.	Below r.v.	Mean (SD)	Above r.v.	Below r.v.	Mean (SD)	Above r.v.	Below r.v.
TSH (microU/mL)	2.3 ± 1.4	2.3%	6.2%	2.6 ± 1.2	11.1%	0%	3.1 ± 2.1	0%	14.3%
fT3 (pg/mL)	2.7 ± 0.7	25.5%	0%	2.4 ± 0.4	57.1%	0%	2.9 ± 0.8	28.6%	0%
fT4 (pg/mL)	8.0 ± 2.0	2.6%	5.2%	8.5 ± 3.7	0%	14.3%	7.2 ± 2.8	25%	12.5%
Na (mmol/L)	139.8 ± 2.8	4.2%	1.4%	139.9 ± 2.7	2.4%	0%	140.5 ± 2.4	0.9%	2.8%
K (mmol/L)	4.2 ± 0.4	0.7%	0.7%	4.3 ± 0.3	1.2%	0%	4.3 ± 0.4	9.9%	0.9%
Cl (mmol/L)	101.6 ± 3.2	11.8%	0%	102.6 ± 2.7	1.2%	1.2%	103.3 ± 2.4	1%	0%
P (mg/dL)	3.9 ± 0.5	0.7%	10.6%	4.1 ± 0.6	7.3%	8.5%	4.2 ± 0.4	0%	16.3%
Mg (mg/dL)	2.2 ± 0.2	0%	0%	2.0 ± 0.2	0%	0%	2.1 ± 0.1	0%	0%
Ca (mg/dL)	9.9 ± 0.5	0%	12.7%	9.7 ± 0.4	0%	0%	9.7 ± 0.4	0%	1%

## Data Availability

The data presented in this study are available on request from the corresponding author due to legal restrictions, i.e., personal and medical data protection laws.
